# Novel genetic variants in the NLRP3 inflammasome-related *PANX1* and *APP* genes predict survival of patients with hepatitis B virus-related hepatocellular carcinoma

**DOI:** 10.1007/s12094-024-03634-x

**Published:** 2024-08-01

**Authors:** Yingchun Liu, Yuman Fan, Rongbin Gong, Moqin Qiu, Xiaoxia Wei, Qiuling Lin, Zihan Zhou, Ji Cao, Yanji Jiang, Peiqin Chen, Bowen Chen, Xiaobing Yang, Yuying Wei, RuoXin Zhang, Qiuping Wen, Hongping Yu

**Affiliations:** 1 Department of Experimental Research, Guangxi Medical University Cancer Hospital, 71 Hedi Road, Nanning, Guangxi China; 2 Key Cultivated Laboratory of Cancer Molecular Medicine of Guangxi Health Commission, Guangxi Medical University Cancer Hospital, 71 Hedi Road, Nanning, Guangxi China; 3 Department of Epidemiology and Health Statistics, School of Public Health, Guangxi Medical University, 22 Shuangyong Road, Nanning, Guangxi China; 4 Department of Respiratory Oncology, Guangxi Medical University Cancer Hospital, 71 Hedi Road, Nanning, Guangxi China; 5 Department of Clinical Research, Guangxi Medical University Cancer Hospital, 71 Hedi Road, Nanning, Guangxi China; 6 Department of Cancer Prevention and Control, Guangxi Medical University Cancer Hospital, 71 Hedi Road, Nanning, Guangxi China; 7 Department of Scientific Research, Guangxi Medical University Cancer Hospital, 71 Hedi Road, Nanning, Guangxi China; 8 Editorial Department of Chinese Journal of Oncology Prevention and Treatment, Guangxi Medical University Cancer Hospital, 71 Hedi Road, Nanning, Guangxi China; 9 Department of Epidemiology, School of Public Health, Key Laboratory of Public Health Safety, Ministry of Education, Fudan University, Shanghai, China; 10 Key Laboratory of Early Prevention and Treatment for Regional High Frequency Tumor (Guangxi Medical University), Ministry of Education, 22 Shuangyong Road, Nanning, Guangxi China; 11 Guangxi Key Laboratory of Early Prevention and Treatment for Regional High Frequency Tumor, Nanning, 22 Shuangyong Road, Guangxi China

**Keywords:** HBV-related hepatocellular carcinoma, NLRP3 inflammasome, Genetic variants, Overall survival

## Abstract

**Background:**

The nod-like receptor protein 3 (NLRP3) is one of the most characterized inflammasomes involved in the pathogenesis of several cancers, including hepatocellular carcinoma (HCC). However, the effects of genetic variants in the NLRP3 inflammasome-related genes on survival of hepatitis B virus (HBV)-related HCC patients are unclear.

**Methods:**

We performed multivariable Cox proportional hazards regression analysis to evaluate associations between 299 single-nucleotide polymorphisms (SNPs) in 16 NLRP3 inflammasome-related genes and overall survival (OS) of 866 patients with HBV-related HCC. We further performed expression quantitative trait loci (eQTL) analysis using the data from the GTEx project and 1000 Genomes projects, and performed differential expression analysis using the TCGA dataset to explore possible molecular mechanisms underlying the observed associations.

**Results:**

We found that two functional SNPs (*PANX1* rs3020013 A > G and *APP* rs9976425 C > T) were significantly associated with HBV-related HCC OS with the adjusted hazard ratio (HR) of 0.83 [95% confidence interval (CI) = 0.73–0.95, *P* = 0.008], and 1.26 (95% CI = 1.02–1.55, *P* = 0.033), respectively. Moreover, the eQTL analysis revealed that the rs3020013 G allele was correlated with decreased mRNA expression levels of *PANX1* in both normal liver tissues (*P* = 0.044) and whole blood (*P* < 0.001) in the GTEx dataset, and *PANX1* mRNA expression levels were significantly higher in HCC samples and associated with a poorer survival of HCC patients. However, we did not observe such correlations for *APP* rs9976425.

**Conclusions:**

These results indicated that SNPs in the NLRP3 inflammasome-related genes may serve as potential biomarkers for HBV-related HCC survival, once replicated by additional larger studies.

**Supplementary Information:**

The online version contains supplementary material available at 10.1007/s12094-024-03634-x.

## Introduction

Liver cancer is one of the most common malignancies characterized by the high incidence and mortality. According to recent cancer burden data for China, there were 367,700 new cases of liver cancer and 316,500 deaths in 2022 in China [1]. Histologically, about 75–85% of liver cancer patients are classified as hepatocellular carcinoma (HCC) [2]. Hepatitis B virus (HBV) infection is the leading cause of HCC, and 87.7% of HCC patients have been infected with HBV in Southern China [3]. Most patients are diagnosed at the middle and advanced stages, and the 5-year survival rate is only 12.1% [4]. Currently, surgery is one of the most effective treatments for patients with an early stage HCC, with a 5-year survival rate of more than 70% [5, 6]. Nevertheless, patients with similar clinical characteristics and treatment may have a different survival, which suggests that genetic variants may have an effect on survival of HBV-related HCC patients. Single-nucleotide polymorphisms (SNPs), the most common type of genetic variants, were reported to be associated with survival of HBV-related HCC patients possibly by affecting gene expression and function [7, 8].

Inflammasome, an intracellular multi-protein complex, is a macromolecular platform formed in response to damage-associated and pathogen-associated molecular patterns, and the formation of inflammasome will lead to interleukin-1 secretion and pyroptosis [9]. Studies have shown that inflammasome may play a key role in the development of tumors by promoting their proliferative invasion and angiogenesis [10–14]. The nod-like receptor protein 3 (NLRP3) is one of the most characterized inflammasome [15], and over-activation of the NLRP3 inflammasome induces an excessive immune-inflammatory response [16]. One study showed that under oxidative stress, NLRP3 inflammasome-mediated inflammation and pyroptosis were enhanced through upregulating mitoROS production by HBV X protein, which played an important role in the development and progression of HBV-related HCC [17]. In addition, the upregulation of the NLRP3 inflammasome effectively inhibits HCC cell invasion and migration [18].These findings indicate that NLRP3 inflammasome may play an important role in the progression of HBV-related HCC.

Several genome-wide association studies (GWASs) of HCC were reported in recent years [19–21], but these studies mainly focused on the susceptibility and those genetic variants that reached a genome-wide significance. In the pathway-based analysis strategy, the stringent significance threshold in GWAS can be substantially relaxed due to the reduction in the number of SNPs to be tested [22]. In the post-GWAS era, this pathway-based analysis strategy can be applied to prioritize genes for identifying potentially functional SNPs and for additional functional investigations [23]. To date, SNPs in the NLRP3 inflammasome-related genes have been found to act as predictors for survival of patients with chronic myeloid leukemia and non-small cell lung cancer [24, 25], but the role of these SNPs in survival of HCC is still unclear. Therefore, we hypothesized that SNPs in the NLRP3 inflammasome-related genes were associated with survival of patients with HBV-related HCC. In the current study, we tested this hypothesis by performing multivariable Cox proportional hazards regression analysis with a study population of patients with HBV-related HCC after hepatectomy.

## Materials and methods

### Study populations

The current study included a total of 866 HCC patients who were seropositive for HBV and underwent hepatectomy in Guangxi Medical University Cancer Hospital between July 2007 and December 2017. Patients with anti-HCV seropositivity and receiving preoperative anti-tumor therapy were excluded, and the specific inclusion and exclusion criteria were described in a previous publication [7]. Patients with an operable HCC were defined as Barcelona Clinical Liver Cancer (BCLC) stage, and some patients of BCLC B or C stages were included according to the additional criteria of a Chinese Guideline for surgical treatment [2].

Patients’ demographic and clinical characteristics, including age, sex, smoking status, drinking status, AFP levels, cirrhosis, embolus, and BCLC stage, were collected and used as covariables for further statistical analyses. Furthermore, a sample of 5-mL of peripheral blood was collected from the patients for DNA extraction at the time of enrollment. For the first two years after hepatectomy, patients were followed up every 3 months, and then every 6 months in the following year. Overall survival (OS) refers to the time between hepatectomy and last follow-up or death with the last follow-up time in March 2020. An informed consent form was approved by the Institutional Review Board of Guangxi Medical University Cancer Hospital (LW2024012).

### Genotyping, genes and SNPs selection

Genotyping was performed using Illumina Infinium® Global Screening Assay (GSA, GSAMD-24v1-0, Illumina, San Diego) at Genenergy Biotechnology (Shanghai, China) using the Illumina iScan System according to the manufacturer’s instructions. The genes involved in the NLRP3 inflammasome were selected by the Molecular Signatures Database (https://www.gsea-msigdb.org/gsea/msigdb/search.jsp) with a keyword ‘NLRP3’, from which 16 candidate genes were included for further analyses. These 16 candidate genes were *NFKB2, P2RX7, HSP90AB1, HMOX1, MEFV, PYCARD, NFKB1, PANX1, TXN, CASP1, PSTPIP1, APP, NLRP3, SUGT1, RELA, and TXNIP* (Table S1). First, we matched the candidate genes from homo sapiens (human) genome assembly GRCh37 (hg19) for their chromosomal positions, and extracted the genotype data of SNPs in these genes and the corresponding 2-kb upstream and downstream regions from the Han Chinese in Beijing (CHB) in the 1000 Genomes Project (http://www.internationalgenome.org/home). Quality control of the SNPs was based on the following criteria: (a) minor allele frequency (MAF)  >  0.05; (b) Hardy–Weinberg equilibrium test *P* value  >  10^–6^; and (c) individual call rate  >  95%. Second, we used RegulomeDB (http://www.regulomedb.org) to select potentially functional SNPs (rank ≤ 1f). Lastly, tagging SNPs were identified using linkage disequilibrium (LD) analysis (*r*^2^ ≥ 0.8) by the Haploview 4.2 software.

### Statistical analysis

We assessed population stratification by principal component analysis (PCA). Multivariable Cox proportional hazards regression analysis was used to assess associations between SNPs in the NLRP3 inflammasome-related genes and HBV-related HCC OS, with adjustment for the above-mention covariables using the GenABEL package of R software [26]. Then, Bayesian false-discovery probability (BFDP) and false-positive report probability (FPRP) were used to reduce the possibility of potential false-positive results, and SNPs with a BFDP < 0.8 or FPRP < 0.20 were included in subsequent analyses [27, 28]. We further performed stepwise multivariable Cox regression analysis to identify independent SNPs, and performed the expression quantitative trait loci (eQTL) analysis to explore correlations between independent SNPs and mRNA expression levels of corresponding genes in the Genotype-Tissue Expression (GTEx) project (https://www.gtexportal.org/home/datasets) and 1000 Genomes project. Meanwhile, The Cancer Genome Atlas (TCGA) database was used to examine differential mRNA expression levels of genes between HCC tissues and adjacent normal tissues. Furthermore, the Kaplan–Meier (KM) survival curve was used to visualize the associations between mRNA expression of genes and survival of patients with liver cancer from KM plotter (http://kmplot.com). RegulomeDB 2.2 (http://regulome.stanford.edu/), SNPinfo (https://manticore.niehs.nih.gov/snpinfo/snpfunc.html), and HaploReg 4.2 (https://pubs.broadinstitute.org/mammals/haploreg/haploreg.php) were used to explore the potential function of SNPs. The cBioPortal for Cancer Genomics (http://www.cbioportal.org) database was performed to examine mutation status of identified genes in HCC.

All statistical analyses were accomplished by PLINK (version 1.09) and R software (version 3.2.3 and 4.1.2), and it was considered statistically significant for *P* < 0.05.

## Results

### Associations of SNPs in the NLRP3 inflammasome-related genes with OS of HBV-related HCC patients

**Fig. 1:**
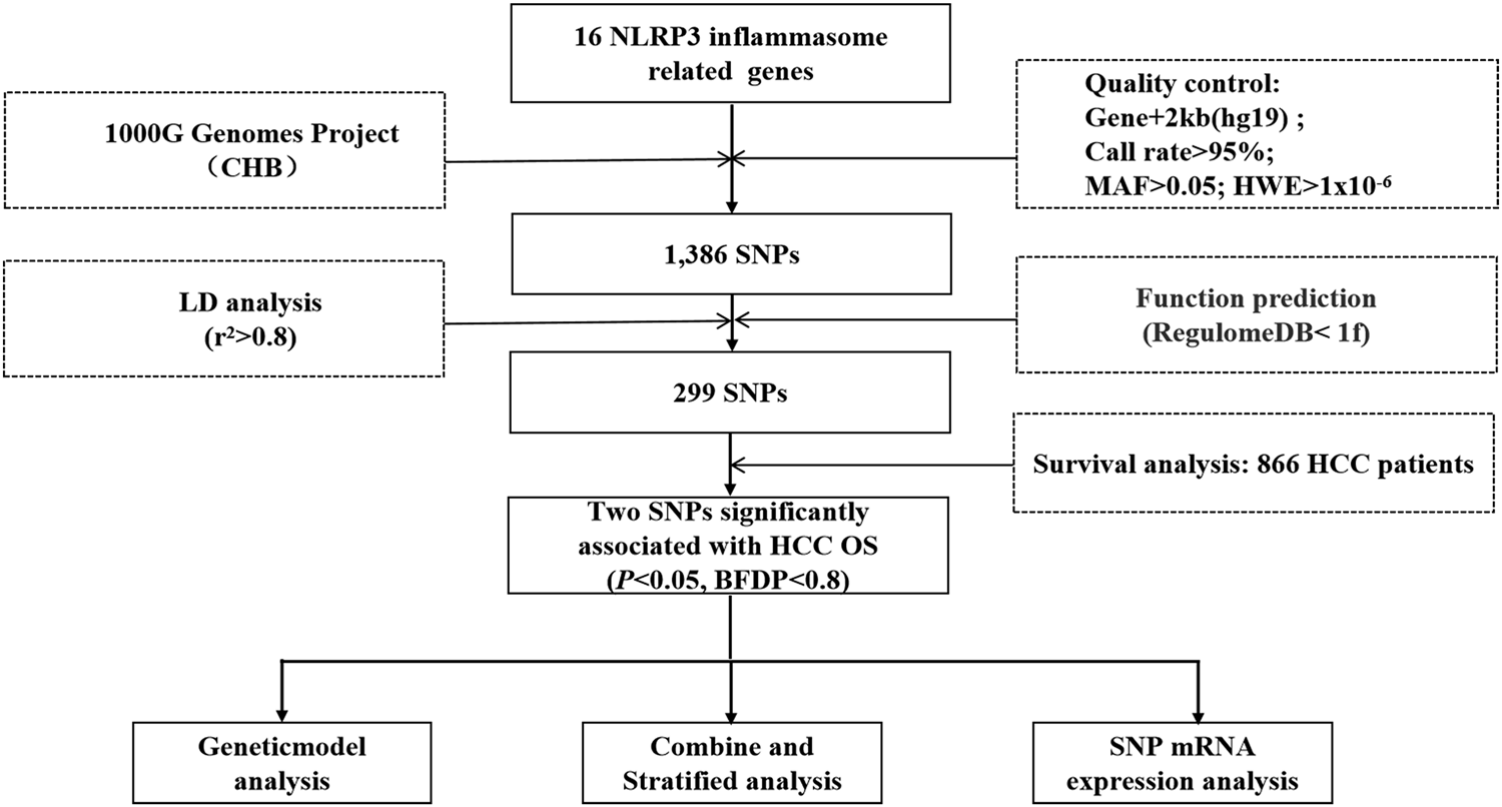
Work flowchart of study design. *CHB* Chinese Han population in Beijing, *MAF* minor allele frequency, *HWE* Hardy–Weinberg equilibrium, *SNP* single-nucleotide polymorphism, *LD* linkage disequilibrium, *HCC* hepatocellular carcinoma, *OS* over survival, *BFDP* Bayesian false-discovery probability

The basic characteristics of 866 HBV-related HCC patients are described in Table S2. The results of PCA showed that none of the first ten principal components were significantly associated with HBV-related HCC OS (Table S3), indicating that there was no noticeable population stratification in the subjects. The detail flowchart of the current study is depicted in Fig. 1. First, we extracted a total of 1386 SNPs in 16 NLRP3 inflammasome-related genes from the 1000 Genomes Project. Then, the functional prediction and LD analysis resulted in the retention of only 299 SNPs for subsequent association analysis. Finally, two SNPs (*PANX1* rs3020013 A > G and *APP* rs9976425 C > T) were identified to be significantly associated with HBV-related HCC OS (*P* < 0.05, BFDP < 0.8) (Table 1).

**Table 1:** Associations of two validated significant SNPs with HBV-related HCC OS

SNP	Chr	Gene	Position	MAF	Allele^a^	HR (95% CI)	BFDP	FPRP	*P*^b^
rs3020013	11	*PANX1*	93,909,540	0.488	A > G	0.83 (0.73–0.95)	0.581	0.058	0.008
rs9976425	21	*APP*	27,267,546	0.116	C > T	1.26 (1.02–1.55)	0.778	0.214	0.033

*SNP* single-nucleotide polymorphism, *OS* overall survival, *HBV-related HCC* hepatitis B virus-related hepatocellular carcinoma, *Chr* chromosome, *MAF* minor allele frequency, *BFDP* Bayesian false-discovery probability, *FPRP* false-positive report probability, *BCLC* Barcelona clinic liver cancer

^a^Major allele > minor allele

^b^Multivariable Cox regression analyses were adjusted for age, sex, smoking status, drinking status, AFP level, Cirrhosis, embolus, and BCLC stage

We then preformed stepwise Cox regression analysis with adjustment for covariables to further identify independent predictors of OS in patients with HBV-related HCC, and *PANX1* rs3020013 and *APP* rs9976425 were identified as the independent SNPs (Table S4). Furthermore, we analyzed the effects of *PANX1* rs3020013 and *APP* rs9976425 on HBV-related HCC OS in different genetic models. As displayed in Table 2, patients with the *PANX1* rs3020013 G allele had a significantly reduced death risk (*P*_trend_ = 0.008), whereas those with the *APP* rs9976425 T allele had an increased risk of death (*P*_trend_ = 0.033). Compared with the AA and GA genotypes, patients with *PANX1* rs3020013 GG genotypes had a better OS (AA + GA vs. GG: hazard ratio (HR) = 0.70, 95% confidence interval (CI) = 0.55–0.89, and *P* = 0.004) in the recessive genetic model, whereas, compared with the CC genotypes, patients with *APP* rs9976425 TC + TT genotypes had a poorer OS (CC vs. TC + TT: HR = 1.27, 95% CI = 1.00–1.60, and *P* = 0.047) in the dominant genetic model.

**Table 2:** Associations between two functional SNPs and overall survival of HBV-related HCC patients

Genotype	All	Death (%)	Univariable analysis		Multivariable analysis	
			HR (95% CI)	*P*	HR (95% CI)	*P*^a^
*PANX1* rs3020013 A > G						
AA	223	124 (55.6)	1.00		1.00	
GA	441	210 (47.6)	0.85 (0.68–1.06)	0.156	0.95 (0.76–1.19)	0.662
GG	202	85 (42.1)	0.69 (0.50–0.91)	0.008	0.68 (0.51–0.90)	0.007
Trend test						0.008
GA + GG	643	295 (45.9)	0.80 (0.65–0.98)	0.035	0.85 (0.69–1.05)	0.141
AA + GA	664	334 (50.3)	1.00		1.00	
GG	202	85 (42.1)	0.76 (0.60–0.97)	0.027	0.70 (0.55–0.89)	0.004
*APP* rs9976425 C > T						
CC	677	322 (47.6)	1.00		1.00	
TC	187	90 (48.1)	1.16 (0.92–1.47)	0.212	1.24 (0.98–1.58)	0.074
TT	12	7 (58.3)	1.53 (0.72–3.24)	0.266	1.68 (0.79–3.58)	0.178
Trend test						0.033
TC + TT	189	97 (51.3)	1.18 (0.94–1.48)	0.151	1.27 (1.00–1.60)	0.047
CC + TC	854	412 (48.2)	1.00		1.00	
TT	12	7 (58.3)	1.48 (0.70–3.14)	0.301	1.59 (0.75–3.39)	0.226
NUG^b^						
0	154	61 (39.6)	1.00		1.00	
1	571	285 (49.9)	1.36 (1.03–1.79)	0.032	1.51 (1.14–2.00)	0.004
2	141	73 (51.8)	1.58 (1.13–2.23)	0.008	1.87 (1.32–2.65)	< 0.001
Trend test						< 0.001
0	502	61 (45.4)	1.00		1.00	
1–2	264	358 (72.3)	1.40 (1.06–1.84)	0.017	1.57 (1.19–2.07)	0.001

*SNP* single-nucleotide polymorphism, *HCC* hepatocellular carcinoma, *HR* hazards ratios, *CI* confidence interval, *BCLC* Barcelona clinic liver cancer, *NUG* number of unfavorable genotypes

^a^Multivariable Cox regression analyses were adjusted for age, sex, smoking status, drinking status, AFP level, Cirrhosis, embolus, and BCLC stage

^b^Unfavorable genotypes were *PANX1* rs3020013 AA + GA and *APP* rs9976425 TC + TT

### Functional analysis

To assess biological functions of these two independent SNPs, we applied three bioinformatics tools (i.e., RegulomeDB 2.2, SNPinfo, and HaploReg 4.2). As shown in Table S5, both *PANX1* rs3020013 and *APP* rs9976425 showed a rank of 1f in the RegulomeDB. Based on the prediction by HaploReg, we also found that both SNPs might alter protein motifs and potentially affect enhancer histone marks. However, there was no obvious evidence for functional relevance of both SNPs in the SNPinfo database.

### Combined and stratified analysis of the independent SNPs

**Fig. 2:**
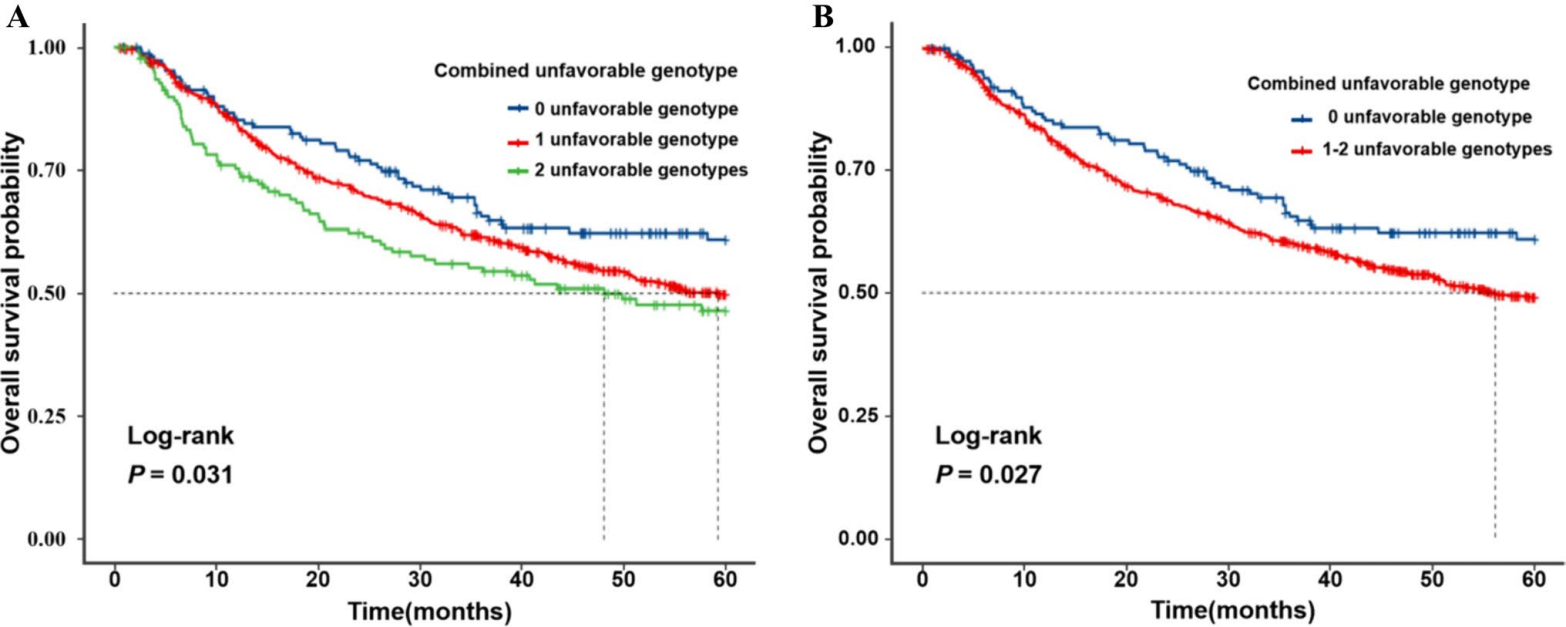
Kaplan–Meier survival curves for HBV-related HCC patients by the combined unfavorable genotypes (i.e., *PANX1* rs3020013 AA and GA, *APP* rs9976425 TC and TT). Kaplan–Meier survival curves HBV-related HCC patients by 0, 1, and 2 unfavorable genotypes groups (**A**); the dichotomized 0 unfavorable genotype group and 1–2 unfavorable genotypes group (**B**). *HBV-related HCC* hepatitis B virus-related hepatocellular carcinoma

To provide a better estimation of the accumulative effect of significant SNPs on survival of HBV-related HCC patients, we combined unfavorable genotypes (i.e., *PANX1* rs3020013 AA + GA and *APP* rs9976425 TC + TT) into a genetic score to divide all patients into three groups based on the number of unfavorable genotypes (NUG) (0, 1, and 2 unfavorable genotypes). As shown in Table 2**,** multivariable Cox regression analysis revealed that an increased genetic unfavorable score was significantly associated with a poorer survival (*P*_trend_ < 0.001). Then, we further dichotomized all patients into two groups according to the NUG score: 0 unfavorable genotype and 1–2 unfavorable genotypes. Compared with patients 0 unfavorable genotypes, those with 1–2 unfavorable genotypes had a significant higher risk of death (HR = 1.57, 95% CI = 1.19–2.07, and *P* = 0.002). Moreover, the Kaplan–Meier survival curves to visualize associations between the unfavorable genotypes and OS in HBV-related HCC are depicted in Fig. 2.

We further performed stratified analysis to evaluate whether the effect of NUG on HBV-related HCC OS was modified by covariables. As shown in Table 3, compared with patients with no NUGs, those patients with one or two NUGs had a worse OS in the subgroup of aged ≤ 47, male, never smokers, never drinkers, with AFP level > 400 ng/mL, with cirrhosis, no embolus, and with BCLC stage B/C (*P* < 0.05 for all). However, no significant interactions were found among all subgroups.

**Table 3:** Stratification analysis of combined unfavorable genotypes with OS of HBV-related HCC patients

Characteristics	0 NUGs		1–2 NUGs		Multivariable analysis		
	All	Death (%)	All	Death (%)	HR (95% CI)	*P*^a^	*P*_inter_^b^
Age (years)							0.730
≤ 47	78	35 (51.3)	356	198 (56.6)	1.59 (1.09–2.31)	0.015	
> 47	76	26 (40.2)	356	160 (47.6)	1.48 (0.97–2.26)	0.067	
Sex							0.935
Female	23	7 (36.8)	83	35 (44.7)	1.43 (0.58–3.49)	0.437	
Male	131	54 (57.2)	629	323 (58.2)	1.51 (1.13–2.03)	0.005	
Smoking status							0.122
No	105	38 (46.3)	440	230 (53.5)	1.83 (1.29–2.59)	< 0.001	
Yes	49	23 (43.7)	272	128 (51.0)	1.15 (0.73–1.81)	0.536	
Drinking status							0.766
No	117	45 (45.3)	497	247 (50.8)	1.60 (1.16–2.21)	0.004	
Yes	37	16 (45.6)	215	111 (56.1)	1.41 (0.82–2.43)	0.509	
AFP level (ng/mL)							0.536
≤ 400	96	35 (41.0)	426	197 (49.3)	1.40 (0.97–2.02)	0.076	
> 400	58	26 (52.3)	286	161 (57.0)	1.71 (1.12–2.60)	0.013	
Cirrhosis							0.237
No	62	26 (45.4)	328	158 (49.2)	1.45 (0.95–2.23)	0.085	
Yes	92	35 (45.5)	384	200 (55.3)	1.74 (1.20–2.52)	0.004	
Embolus							0.827
No	111	35 (36.4)	265	525 (47.2)	1.64 (1.14–2.37)	0.008	
Yes	43	26 (71.0)	187	133 (66.7)	1.45 (0.94–2.22)	0.090	
BCLC stage							0.099
0/A	73	22 (30.4)	354	124 (39.7)	1.10 (0.70–1.74)	0.682	
B/C	81	39 (60.6)	358	234 (64.2)	1.81 (1.28–2.56)	< 0.001	

*OS* overall survival, *HCC* hepatocellular carcinoma, *NUG* number of unfavorable genotypes, *HBV-related HCC* hepatitis B virus-related hepatocellular carcinoma, *HR* hazards ratios, *CI* confidence interval, *BCLC* Barcelona clinic liver cancer

^a^Multivariable Cox regression analyses were adjusted for age, sex, smoking status, drinking status, AFP level, Cirrhosis, embolus, and BCLC stage

^b^*P* for interaction analysis between variables and NUG

### eQTL analysis and differential mRNA expression analysis

**Fig. 3:**
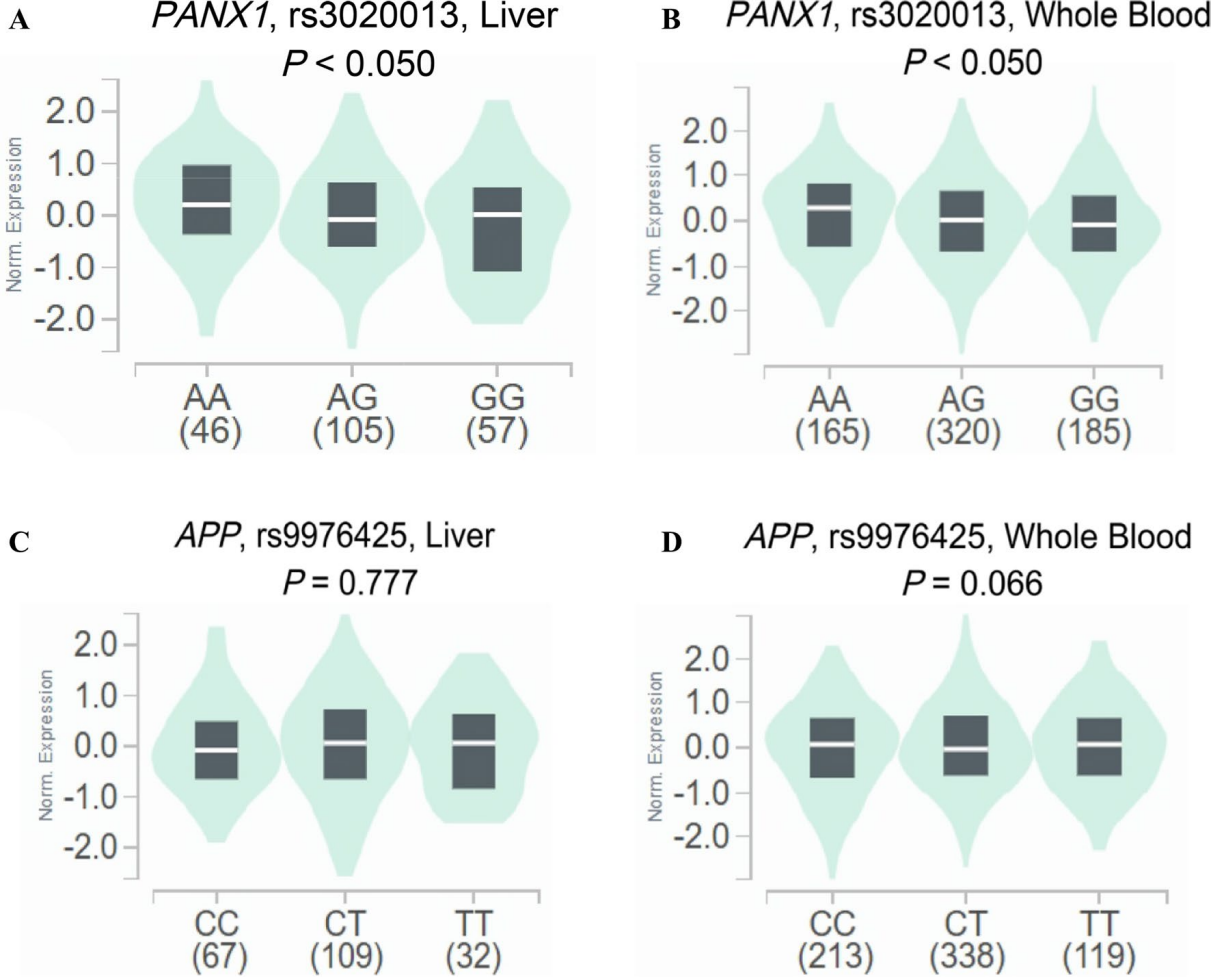
eQTL analysis of *PANX1* rs3020013 and *APP* rs9976425 from GTEx database. The correlation of rs3020013 genotypes and *PANX1* mRNA expression in normal liver tissues (*n* = 208, *P* = 0.045) (**A**) and in whole blood samples (*n* = 670, *P* < 0.001) (**B**); the correlation of rs9976425 genotypes and *APP* mRNA expression in normal liver tissues (*n* = 208, *P* = 0.777) (**C**) and in whole blood samples (*n* = 670, *P* = 0.066) (**D**). *eQTL* expression quantitative trait loci, *GTEx* Genotype-Tissue Expression project

To further explore potential functions of two significant SNPs, we performed the eQTL analysis to identify the correlations between genotypes of two SNPs (i.e., *PANX1* rs3020013 and *APP* rs9976425) and mRNA expression levels of their corresponding genes in 208 normal liver tissues and 670 whole blood samples from the GTEx project and in lymphoblastoid cells from 76 CHB in 1000 Genomes Project. We found that the rs3020013 G allele was correlated with reduced mRNA expression levels of PANX1 in normal liver tissues (*P* = 0.044, Fig. 3A) and whole blood samples (*P* < 0.001, Fig. 3B) from the GTEx project, but such correlations were not observed in additive (*P* = 0.358, Figure S1A), dominant (*P* = 0.670, Figures S1B), and recessive models (*P* = 0.150, Figure S1C) from the 1000 Genomes Project. There was no significant correlation between rs9976425 T allele and mRNA expression levels of *APP* in normal liver tissues (*P* = 0.777, Fig. 3C) and whole blood samples (*P* = 0.060, Fig. 3D) from the GTEx project.

**Fig. 4:**
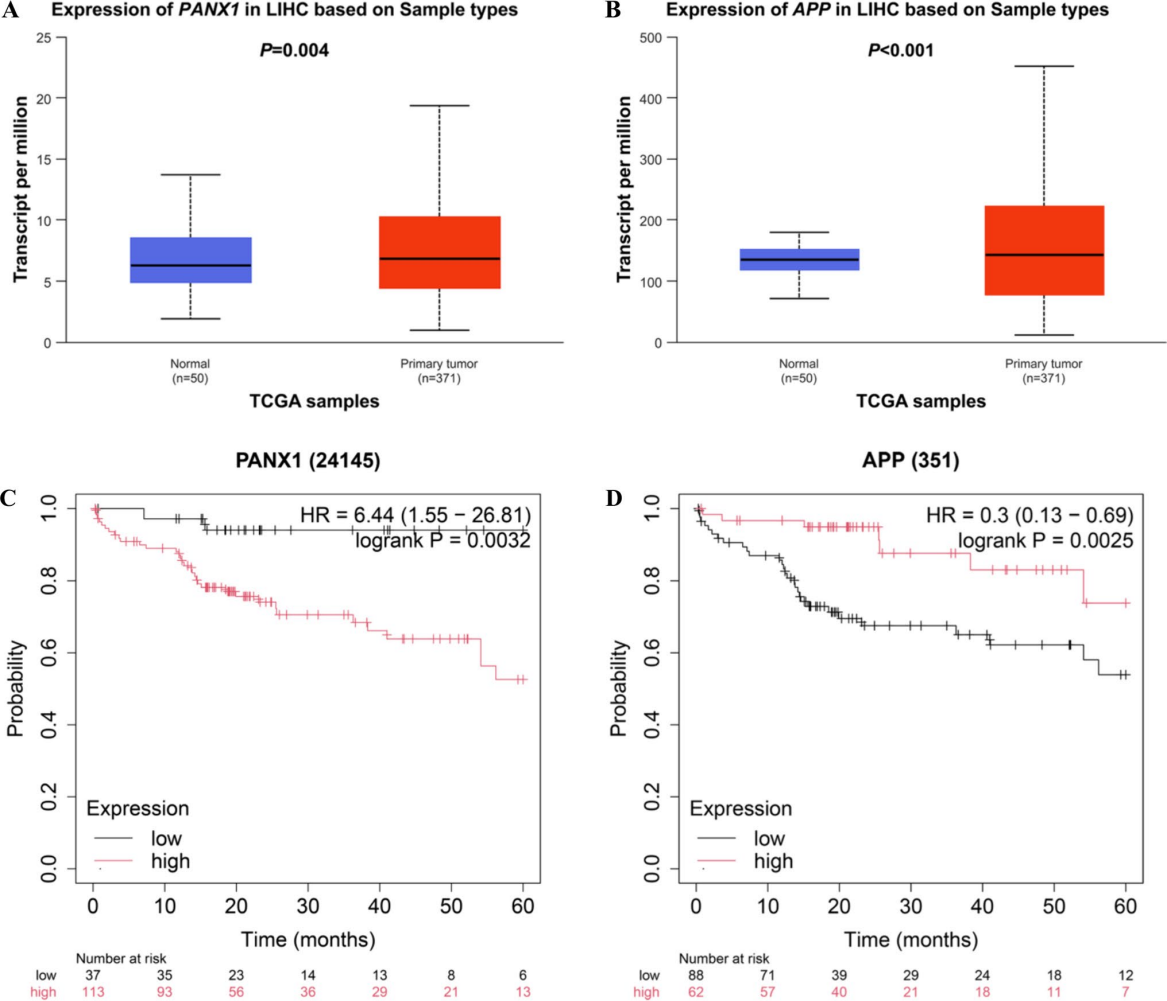
Compared with the normal tissues, higher mRNA expression levels of *PANX1* were found in the HCC tissues from the TCGA database (**A**); higher mRNA expression levels of *APP* were found in the HCC tissues than in the normal tissues in the TCGA database (**B**); higher mRNA expression levels of *PANX1* were significantly correlated with a worse liver cancer survival (**C**); lower mRNA expression levels of *APP* were significantly correlated with a worse liver cancer survival (**D**) *LIHC* Liver Hepatocellular Carcinoma, *HR* hazards ratio, *TCGA *The Cancer Genome Atlas

To estimate associations of the *PANX1* and *APP* genes with the survival of HBV-related HCC, we first compared mRNA expression levels of *PANX1* and *APP* between HCC tissues and adjacent normal tissue obtained from the TCGA database. As shown in Fig. 4A and B, compared with adjacent normal tissues, both the mRNA expression levels of *PANX1* and *APP* were higher in HCC tissues (*P* = 0.004 and *P* < 0.001, respectively).

Additionally, we also evaluated associations between mRNA expression levels of *PANX1* and *APP* and OS of patients with liver cancer in the KM plotter, and found that patients with lower mRNA expression levels of *PANX1* had a better liver cancer OS (HR = 6.44; 95% CI = 1.55–26.81; log-rank *P* = 0.003, Fig. 4C); and higher of mRNA expression levels of *APP* had a better liver cancer OS (HR = 0.30; 95% CI = 0.13–0.69; log-rank *P* = 0.003, Fig. 4D).

### Mutation analysis

Because frequently mutated genes in tumor tissues may have a much greater effect on patients’ survival than SNPs in the same genes, we further investigated the mutation status of *PANX1* and *APP* in liver tumor tissues using the public database of the cBioPortal for Cancer Genomics. As shown in Figure S2A, *PANX1* had a much low somatic mutation rate in different HCC datasets (0.82% in MERiC/Basel, Nat Commun, 2022 [29]; 0.54% in TCGA, Firehose Legacy; and 0.27% in TCGA, PanCancer Atlas) [30], and *APP* also displayed a low mutation rate in different HCC datasets (1.64% in TCGA, PanCancer Atlas [30]; 1.61% in TCGA, Firehose Legacy; 0.82% in MERiC/Basel Nat Commun. 2022 [29]; and 0.43% in AMC Hepatology 2014 [31], Figure S2B). Such low mutation frequencies in both *PANX1* and *APP* are unlikely to have a significant effect on the expression levels of these two genes in HCC.

## Discussion

In the current study, we found two novel SNPs (i.e., *PANX1* rs3020013 A > G and *APP* rs9976425 C > T) in the NLRP3 inflammasome-related genes were significantly associated with OS of patients with HBV-related HCC. Further combined analysis indicated that patients with unfavorable genotypes of both SNPs (*PANX1* rs3020013 AA + GA, *APP* rs9976425 TC + TT) had a worse HBV-related HCC OS. Additionally, the eQTL analysis revealed that the *PANX1* rs3020013 G allele was correlated with decreased *PANX1* mRNA expression levels in normal liver tissues and whole blood samples, although we did not find sufficient evidence for the effect of rs9976425 on *APP* expression.

Pannexin-1 (*PANX1*) gene is located on human chromosome 11q14.3 and consists of five exons and four introns [32]. It has been reported that *PANX1* cleavage and resulting channel activity promoted NLRP3 inflammasome activation during intrinsic and extrinsic apoptosis in macrophages [33, 34]. In lipopolysaccharide (LPS)-treated HK-2 cells, silencing *PANX1* led to the decreased inflammatory cytokines production, and inhibited NLRP3 inflammasome activation and apoptosis [35]. In addition, the decreased *PANX1* mRNA expression in the liver tissue led to the decreased activation of P2X2, and the reduced P2X2 function further decreased the NLRP3-mediated release of interleukin-33 (IL-33) [36]. In addition, *PANX1* may act as an oncogene and is highly expressed in several tumors [37]. Notably, *PANX1* was also upregulated in HCC, and its higher mRNA expression levels were associated with a worse survival of HCC patient and overexpression of *PANX1* promoted HCC cell invasion and migration mainly through regulating epithelial–mesenchymal transition [38]. In the current study, we found that the rs3020013 G allele was associated with a better survival of patients with HBV-related HCC and a lower mRNA expression level of *PANX1*.

The results from bioinformatics tools (i.e., RegulomeDB 2.2 and HaploReg 4.2) provide biological plausibility for the potential function of rs3020013. *PANX1* rs3020013 is marked by enhancer histone markers, possibly interfering with their gene expression through transcriptional regulation. In addition, the mRNA expression levels of *PANX1* were higher in HCC tissues than in adjacent normal tissues, and higher mRNA expression levers of *PANX1* were significantly associated with a poorer HBV-related HCC survival, which is consistent with the previous findings. Thus, the rs3020013 may affect gene function by regulating the mRNA expression levels of *PANX1*. Taken together, these may provide some evidence of the mechanisms underlying the observed association between *PANX1* rs3020013 and HBV-related HCC OS.

Amyloid precursor protein (*APP*) gene, located at 21q21.3 [39], encodes a cell-surface receptor and transmembrane precursor protein that is widely expressed in the liver and pancreas [40]. In transgenic APP/PS1 mice, the animals expressed a human/mouse chimaeric amyloid precursor protein and human presenilin-1, leading to the chronic deposition of amyloid-beta; NLRP3 inflammasome was activated by amyloid-beta in microglia; and in contrast, the deletion of NLRP3 or caspase-1 in these APP/PS1 mice caused microglia to exhibit anti-inflammatory M2 phenotype, with the decreased caspase-1 and IL-1β secretion. [41]. *APP* possesses a wide range of cellular functions, such as the regulation of cellular adhesion, differentiation, and migration [42], which are also essential processes for cancer development. It has been found that the expression level of *APP* is increased in prostate, pancreatic and thyroid cancers [43–46]; when *APP* expression was upregulated, the metastatic ability of tumor cells increased [47], which suggests that it is closely related to tumor growth and metastasis. In the current study, we found that *APP* rs9976425 T allele was associated with a worse survival of patients with HBV-related HCC. However, *APP* mRNA expression in liver cancer tissues was higher than in normal liver tissues from the TCGA dataset, and the results from the online database (http://kmplot.com) showed that higher mRNA expression levels of *APP* were associated with a better survival in patients with liver cancer, for which the small sample sizes of both databases may be a possible explanation. Furthermore, since the available data did not yet support the correlation between the rs9976425 T allele and mRNA expression levels of *APP*, more studies are needed to demonstrate the molecular mechanism underlying the observed associations.

Although we observed some associations between genetic variants in two NLRP3 inflammasome-related genes and HBV-related HCC OS, several limitations in the current study should be discussed. First, we were not able to collect detail information about the treatments, which may not allow us to accurately estimate associations between genetic variants and survival of HBV-related HCC patients in multivariable analyses. Second, because the sample size of the current study was relatively limited, studies with larger HCC patient populations are needed to validate our findings. Finally, although our results indicated that *PANX1* rs3020013 and *APP* rs9976425 may play an important role in HBV-related HCC survival, the exact molecular mechanisms are unclear, and more mechanistic experiments are needed to clarify the underlying molecular mechanisms of *PANX1* rs3020013 and *APP* rs9976425 in the progression of HCC.

## Conclusion

Our finding indicated that both *PANX1* rs3020013 and *APP* rs9976425 in the NLRP3 inflammasome-related genes may serve as potential prognostic factors for HBV-related HCC patients, once replicated by additional studies in lager population.

This project fully considered and protected the rights and interests of the study objects. It meets the criteria of Ethical Review Committee. The Medical Ethics Committee of Guangxi Medical University Cancer Hospital has approved the protocol.

## Supplementary Information

Below is the link to the electronic supplementary material.

Supplementary file1 (DOCX 353 KB)

## Data Availability

Data presented in this study are included in the article/supplementary material. Available at ().
