# Supplementary material for: Novel genetic variants in the NLRP3 inflammasome-related PANX1 and APP genes predict survival of patients with hepatitis B virus-related hepatocellular carcinoma
Source: Clin Transl Oncol. 2024 Aug 1;27(2):630–41. doi: 10.1007/s12094-024-03634-x (PMC11782428; doi:10.1007/s12094-024-03634-x)
Supplement: Supplementary file 1 — Supplementary file1 (DOCX 353 KB) [file 12094_2024_3634_MOESM1_ESM.docx]

**Supplementary Figures and Tables**

**Contents**

**Figure S1.** eQTL analyses for *PANX1* rs3020013 from 1000 Genomes dataset.

**Figure S2.** Mutation frequency of *PANX1* and *APP* in HCC.

**Table S1.** List of NLRP3 inflammasome pathway genes.

**Table S2.** Associations of demographics and clinical characteristics with OS in HBV-related HCC patients.

**Table S3.** Associations of the first 10 PCs and overall survival of HBV-related HCC patients

**Table S4.** Stepwise multivariable Cox regression analysis for the identification of independent SNPs in the NLRP3 inflammasome pathway genes in HBV-related HCC patients.

**Table S5.** Functional annotation of two independent SNPs.

| A | B | C |
| --- | --- | --- |
| 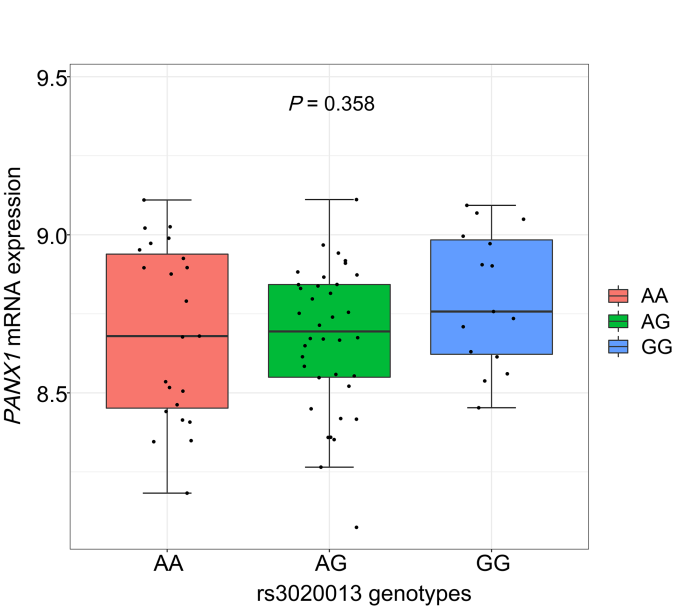 | 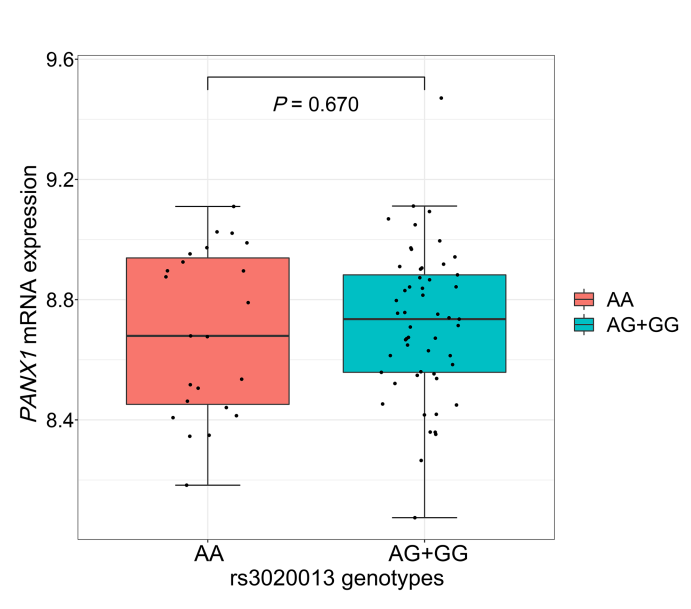 | 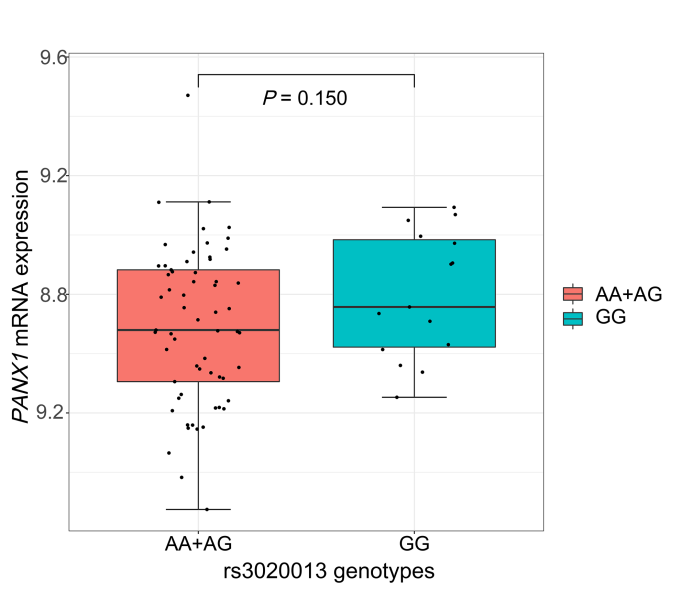 |
| **Figure S1.** The expression quantitative trait locus (eQTL) analysis for *PANX1* rs3020013 in 76 Han Chinese in Beijing from the 1000 Genomes Project. Correlation of rs3020013 with mRNA expression of *PANX1* (A) in addictive model, (B) in dominant model, and (C) in recessive model. | | |

| 1. ***PANX1*** | 1. ***APP*** |
| --- | --- |
| **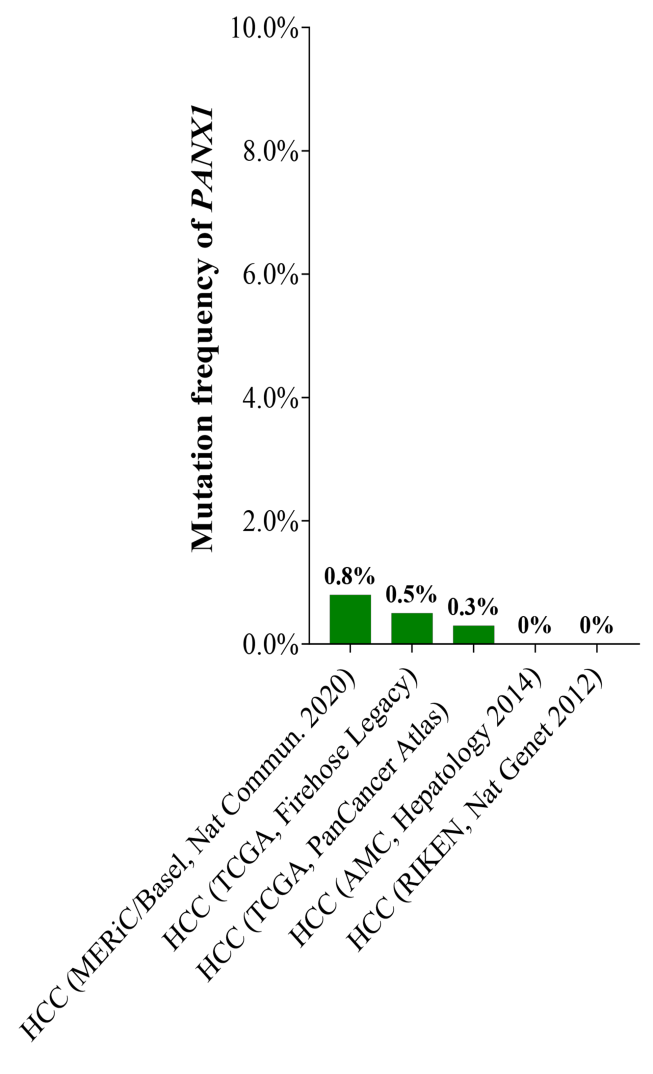** | **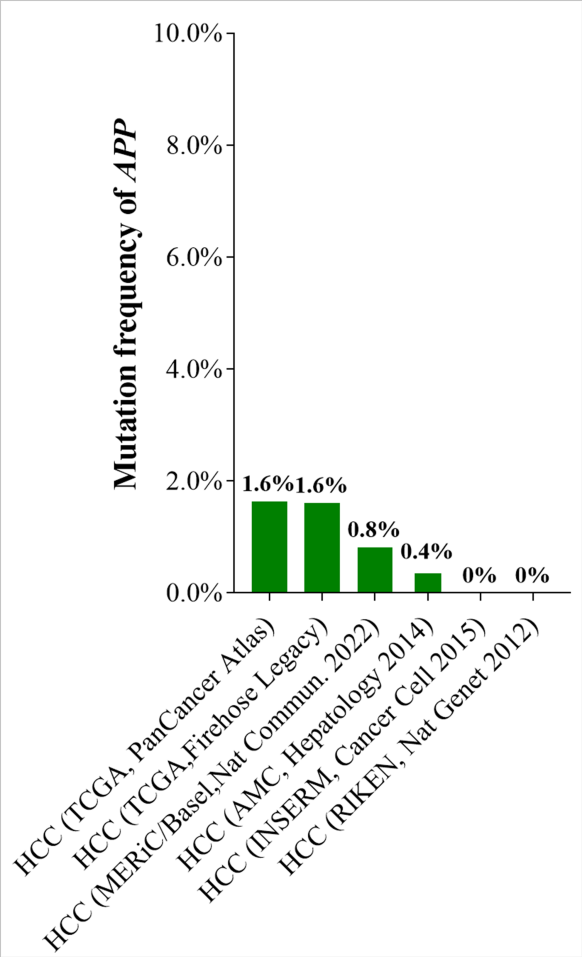** |
| **Figure S2**. Mutation frequency of *PANX1* and *APP* in HCC.  Mutation frequency of *PANX1* **(A)** and *APP* **(B)** in HCC from the database of the cBioPortal for Cancer Genomics(http://www.cbioportal.org).  Abbreviation: HCC, Hepatocellular Carcinoma. | |

| **Table S1**. List of NLRP3 inflammasome pathway genes. | | |  |
| --- | --- | --- | --- |
| Source | Name of gene-set | Selected genes | Number of genes |
| REACTOME_THE_NLRP3_  INFLAMMASOME | The NLRP3 inflammasome | *NFKB2, P2RX7, HSP90AB1, HMOX1, MEFV, PYCARD, NFKB1, PANX1, TXN, CASP1, PSTPIP1, APP, NLRP3, SUGT1, RELA, TXNIP* | 16 |
| Keyword: NLRP3  Organism: Homo sapiens  Website: http://www.gsea-msigdb.org/gsea/msigdb/search.jsp | | | |

| **Table S2.** Associations of demographics and clinical characteristics with OS in HBV-related HCC patients. | | | | |
| --- | --- | --- | --- | --- |
| Characteristics | All | Death (%) | HR (95% CI) | *P ^a^* |
| Age (year) |  |  |  |  |
| ≤ 47 | 434 | 233 (53.7) | 1.00 |  |
| > 47 | 432 | 186 (43.1) | 0.81 (0.66-0.99) | 0.036 |
| Sex |  |  |  |  |
| Female | 106 | 42 (39.6) | 1.00 |  |
| Male | 760 | 377 (49.6) | 1.26 (0.90-1.76) | 0.176 |
| Smoking status |  |  |  |  |
| No | 545 | 268 (49.2) | 1.00 |  |
| Yes | 321 | 151 (47.0) | 0.91 (0.71-1.17) | 0.475 |
| Drinking status |  |  |  |  |
| No | 614 | 292 (47.6) | 1.00 |  |
| Yes | 252 | 127 (50.4) | 1.08 (0.84-1.41) | 0.541 |
| AFP level (ng/ml) |  |  |  |  |
| ≤ 400 | 522 | 232 (44.4) | 1.00 |  |
| > 400 | 344 | 187 (54.4) | 1.29 (1.05-1.57) | 0.015 |
| Cirrhosis |  |  |  |  |
| No | 390 | 184 (47.2) | 1.00 |  |
| Yes | 476 | 235 (49.4) | 1.04 (0.85-1.26) | 0.702 |
| Embolus |  |  |  |  |
| No | 636 | 260 (40.1) | 1.00 |  |
| Yes | 230 | 159 (69.1) | 1.74 (1.37-2.21) | <0.001 |
| BCLC stage |  |  |  |  |
| 0/A | 427 | 146 (34.2) | 1.00 |  |
| B/C | 439 | 273 (62.2) | 1.98 (1.56-2.52) | <0.001 |
| ^a^ Adjusted for age, sex, smoking status, drinking status, AFP level, cirrhosis, embolus, and BCLC stage.  Abbreviation: OS, Overall Survival; HCC, Hepatocellular Carcinoma; HR, Hazards Ratio; CI, Confidence Interval; AFP, Alpha-Fetoprotein; BCLC, Barcelona Clinic Liver Cancer. | | | | |

| **Table S3** Associations of the first 10 PCs and overall survival of HBV-related HCC patients | | | | | |
| --- | --- | --- | --- | --- | --- |
| PC | coef | Exp (coef) | SE (coef) | Z | *P*^a^ |
| PC1 | -0.036 | 0.965 | 1.523 | -0.023 | 0.981 |
| PC2 | -0.887 | 0.412 | 1.450 | -0.612 | 0.541 |
| PC3 | 0.911 | 2.488 | 1.480 | 0.616 | 0.538 |
| PC4 | 0.779 | 2.180 | 1.474 | 0.529 | 0.597 |
| PC5 | 2.552 | 12.842 | 1.489 | 1.726 | 0.084 |
| PC6 | 0.693 | 1.999 | 1 428 | 0.485 | 0.627 |
| PC7 | 1.513 | 4.539 | 1.449 | 1.044 | 0.297 |
| PC8 | 0.158 | 1.171 | 1.487 | 0.105 | 0.915 |
| PC9 | 0.667 | 1.949 | 1.454 | 0.459 | 0.646 |
| PC10 | -0.128 | 0.879 | 1.454 | -0.088 | 0.930 |
| ^a^ Multivariable Cox regression analyses were adjusted for age, sex, smoking status, drinking status, AFP level, Cirrhosis, embolus, BCLC stage, PC1, PC2, PC3, PC4, PC5, PC6, PC7, PC8, PC9 and PC10.  Abbreviations: PC, Principal Component; OS, Overall Survival; HBV-related HCC, Hepatitis B Virus- related Hepatocellular Carcinoma. | | | | | |

| **Table S4.** Stepwise multivariable Cox regression analysis for the identification of independent SNPs in the NLRP3 inflammasome pathway genes in HBV-related HCC patients. | | | | |
| --- | --- | --- | --- | --- |
| Variables | Category | Frequency | HR (95% CI) | Pa |
| Age | ≤47/>47 | 434/432 | 0.80 (0.65-0.98) | 0.033 |
| AFP (ng/mL) | ≤400/>400 | 522/344 | 1.29 (1.04-1.58) | 0.018 |
| Embolus | No/Yes | 636/230 | 1.64 (1.29-2.08) | <0.001 |
| BCLC | 0/A / B/C | 439/427 | 2.32 (1.81-2.99) | <0.001 |
| *PANX1* rs3020013 | AA/GA/GG | 223/441/202 | 0.85 (0.74-0.98) | 0.026 |
| *APP* rs9976425 | CC/TC/TT | 677/187/12 | 1.33 (1.07-1.64) | 0.009 |
| a Adjusted for age, sex, smoking status, drinking status, AFP level, cirrhosis, embolus, and BCLC stage.  Abbreviation: HR, Hazards Ratio; CI, Confidence Interval; AFP, Alpha-Fetoprotein; BCLC, Barcelona Clinic Liver Cancer. | | | | |

| **Table S5.** Functional annotation of two independent SNPs | | | | | | | | | |
| --- | --- | --- | --- | --- | --- | --- | --- | --- | --- |
| SNP | Gene | SNPinfo ^a^ | Regulome  DB rank ^b^ | Haploreg v4.2 ^c^ | | | | | |
|  |  |  |  | Promoter  histone marks | Enhancer  histone marks | DNAse | Motifs  changed | Selected  eQTL hits | dbSNP  Func annot |
| rs3020013 | *PANX1* | - | 1f |  | 6 tissues | KID | RFX5 | 8 hits | intronic |
| rs9976425 | *APP* | - | 1f |  | BLD |  | ERalpha-a, STAT, TLX1::NFIC |  | intronic |
| ^a^ http://snpinfo.niehs.nih.gov/snpinfo/snpfunc.htm;  ^b^ http://www.regulomedb.org/index;  ^c^ <https://pubs.broadinstitute.org/mammals/haploreg/haploreg.php.>  Abbreviation: eQTL, Expression Quantitative Trait Locus. | | | | | | | | | |
